# Symptoms related to new flight attendant uniforms

**DOI:** 10.1186/s12889-017-4982-4

**Published:** 2018-01-03

**Authors:** Eileen McNeely, Steven J. Staffa, Irina Mordukhovich, Brent Coull

**Affiliations:** 1 Department of Environmental Health, Harvard T.H. Chan School of Public Health, Building 1, Room 1401, 655 Huntington Avenue, Boston, MA 02115 USA; 2 Center for Health and the Global Environment, Harvard T.H. Chan School of Public Health, The Landmark Center, 4th Floor West, P.O. Box 15677, 401 Park Drive, Boston, MA 02215 USA

**Keywords:** Environmental health, Textiles, Uniforms, Flight attendants, Occupational epidemiology, Allergic, Respiratory, Dermatological, Multiple chemical sensitivity

## Abstract

**Background:**

Flight attendants at Alaska Airlines reported health symptoms after the introduction of new uniforms in 2011. The airline replaced the uniforms in 2014 without acknowledging harm. To understand possible uniform-related health effects, we analyzed self-reported health symptoms in crew who participated in the Harvard Flight Attendant Health Study between 2007 and 2015, the period before, during, and after the introduction of new uniforms.

**Methods:**

We calculated a standardized prevalence of respiratory, dermatological and allergic symptoms at baseline, as well as during and after uniform changes in 684 flight attendants with a varying number of surveys completed across each time point. We used Generalized Estimating Equations (GEE) to model the association between symptoms at baseline versus the exposure period after adjusting for age, gender and smoking status and weighting respondents for the likelihood of attrition over the course of the study period.

**Results:**

We found the following symptom prevalence (per 100) increased after the introduction of new uniforms: multiple chemical sensitivity (10 vs 5), itchy/irritated skin (25 vs 13), rash/hives (23 vs 13), itchy eyes (24 vs 14), blurred vision (14 vs 6), sinus congestion (28 vs 24), ear pain (15 vs 12), sore throat (9 vs 5), cough (17 vs 7), hoarseness/loss of voice (12 vs 3), and shortness of breath (8 vs 3). The odds of several symptoms significantly increased compared to baseline after adjusting for potential confounders.

**Conclusion:**

This study found a relationship between health complaints and the introduction of new uniforms in this longitudinal occupational cohort.

## What this paper adds

- We know little about the health effects of chemicals in our clothing as compared to substances we ingest, even though skin absorption can be quite efficient and researchers have found metals, dyes, formaldehyde and formaldehyde releasers, dioxin, perfluorinated compounds, flame retardants, phthalates and other plasticizers such as diisodecyclmaleate, pesticides and fungicides in clothing.
- This study offers a unique window into the potential health effects of textile chemicals after the introduction of new work uniforms in an occupational cohort-- a rare opportunity to appreciate a common exposure in a defined population with a specific release date.
- We found significantly increased prevalence of symptoms after the introduction of new uniforms including eye pain/dry eyes/itchy eyes, blurred vision, combined EENT, cough, hoarseness/loss of voice combined lower respiratory, itchy/irritated skin, and rash/hives.
- These findings together with reports of similar health reactions in yet another U.S. flight attendant population after the introduction of new uniforms this year warrants further investigation of the specific chemical toxicants, clothing concentrations, body burdens and health effects.

## Background

In 2011, flight attendants at Alaska Airlines received new uniforms that resulted in 800 health complaints between early 2011 and February 2014, at which point the uniforms were recalled [1]. Employees had reported skin reactions, hair loss, breathing problems, thyroid dysfunction, headaches, fatigue, and chemical sensitivity in association with the rollout of the new uniform. According to the employee union health and safety website for the Association of Flight Attendants-CWA, the airline and the manufacturer conducted separate laboratory tests of the materials, specifically, testing for tributyl phosphate, but found low quantities that were not expected to cause reactions [2]. The flight attendants launched a class action suit against the manufacturer in 2012 but lost the case in 2016 because of a lack of proof of known health consequences at the levels of the chemicals found in the uniforms [3, 4]. In addition, the National Safety and Health Administration (NIOSH) conducted a Health Hazard Review (HHE) and found that the uniforms were unlikely to be related to the health complaints because of a lack of scientific evidence for chemical toxicity according to the quantities in the material and because the prevalence rates of dermatitis in the flight attendants were not dissimilar to the general population [5]. Gauging the health consequences of the new uniforms was complicated by limited data about reactions to textile chemicals in the general population and limited data about the onset and scope of reactions in the flight attendant population specifically.

Further testing of uniform chemicals was ordered by the union and performed by the Hohenstein Textile Testing Institute (Hohenstein, Germany). This laboratory found the presence of several concerning compounds, including dispersion dyes that are banned in the Eurpoepan Union because of suspected carcinogenicity, and tributyl phosphate, a known irritant and potential endocrine disrupting compound [6]. Tributyl phosphate has been found to cause eye, skin, and respiratory irritation if inhaled, in contact with skin or eyes, or ingested [7]. Various heavy metals (i.e. lead, arsenic, cobalt, chromium, and antimony) and biocides (known to be irritants and sensitizers) used to prevent mold growth during shipment were also detected. Despite these findings, a direct connection between exposure to these compounds via textiles, individually or as a mixture, and symptom development in flight attendants or the general population is unknown.

Historically, toxic and hazardous chemical compounds have been found in clothing and furniture since the 1960s [8]. This prompted the EU to create consumer product safety regulations, and the United States to produce voluntary guidelines for product safety [9]. Despite this awareness, case reports of health effects from chemicals in textiles continue to be reported, particularly for synthetic fabrics embedded with anti-wrinkle and stain resistant properties and dyes [10]. In the airline industry alone, TSA agents reported reactions in the eye, ear, nose, and throat as well as dermatological reactions due to new employee uniforms in 2009 [11]. In 2011, the Alaska Airline flight attendants began reporting health symptoms in relation to their new uniforms. Most recently, in late 2016, many American Airlines flight attendants began reporting reactions to their new uniforms sourced from the same uniform supplier as Alaska Airlines [12–14].

Little is known about the prevalence of health effects due to chemical exposures in textiles. In the United States, product labeling does not include chemical treatment information for fabrics [15]. Moreover, the use of chemical compounds in fabric manufacturing is largely unregulated, even though these chemicals include carcinogens, sensitizers, and endocrine disruptors [16].

While the EU requires textile testing, certifying, labeling, and recalling for health protection, U.S. manufacturers follow voluntary guidelines for material testing and chemical reporting that result in a patchwork of practices. Consequently, U.S. consumers may be largely uninformed about chemical exposures in garments and potential health consequences [17].

Today, health complaints related to textiles/clothing remain mostly anecdotal without sufficient information or context to decipher a possible casual relationship. For example, we lack complete information about onset and duration of symptoms, symptom prevalence (including accounts of non-reporting), individual and combined chemicals constituents in clothing, and history of exposure levels in actual use or work conditions. The objective of this study was to explore and quantify the associations between the Alaska Airlines flight attendant health symptoms before, during and after the introduction of new uniforms.

## Methods

We conducted a time series analysis of health symptoms in active Alaska Airline crew who were already part of a larger study, the Harvard Flight Attendant Health Survey. The study began in 2007, with two follow-up periods in 2013 and 2015. The baseline year of 2007 marks the period before the introduction of the uniforms, 2013 (up until February 1, 2014) marks the period when the new uniforms were worn by the flight crew, and the year 2015 marks the period after the uniforms were recalled.

### Study population

The study sample was drawn from all flight attendants who identified as Alaska Airline crew in 2007 and who completed at least one follow up survey of the Harvard Flight Attendant Health Survey. Our cohort consists of all active flight attendants. This selection included 684 Alaska Airlines flight attendants in 2007, 117 flight attendants in 2013, and 249 flight attendants in 2015. There were a total of 65 flight attendants in our sample that completed all three surveys (2007, 2013, and 2015). This subset of flight attendants showed similar results as those found in the full study population.

### Instruments

The Harvard Flight Attendant Survey includes questions about job history (i.e. tenure, work hours, airline, routes, job demands) and work exposures, health symptoms in the last week and last year, medical diagnoses, surgeries, work-related illnesses, and occupational injuries [18]. In addition, we collected demographic and lifestyle-related variables including gender, age, ethnicity, body mass index, smoking status and history of substance use. The health questions were drawn from existing population-based surveys, such as the National Health and Nutrition Examination Survey (NHANES), to allow comparison with the general population. We modified our questions slightly over the years as the format changed from a hard-copy survey in 2007 to an online format in 2013 that allowed the inclusion of more items/questions.

### Variables

#### Health outcomes

Self-reported symptoms noted in each year were considered as health effects of interest. Every survey asked about symptomology in two ways**: “**In the past week, how many days did you experience symptoms?” and “In the past year, did you seek care for symptoms?”. The first question type was recoded as binary(4 or more days vs. 3 or fewer days). The second question type was dichotomized as “yes” or “no”.

We examined associations between uniform exposure and symptoms from the four main impact areas listed below:

1. EENT (Eyes, Ears, Nose, Throat): blurred vision, eye pain/dry eyes/itchy eyes, sinus congestion, ear pain, nosebleeds, sore throat, sinus pain, ear drum rupture, ear infection, runny nose, ringing ears. Note that eye pain, dry eyes, and itchy are were grouped into one variable.
2. Lower Respiratory: shortness of breath, cough, hoarseness/voice loss, wheezing, lung infection (pneumonia) symptoms, asthma symptoms, bronchitis symptoms.
3. Skin: itchy/irritated skin, rash/hives.
4. Multiple Chemical Sensitivity (MCS) symptoms.

First, we created a composite score for each system area by combining multiple symptoms by constructing binary variables to indicate if a flight attendant reported at least one of the symptoms in that impact category. Grouped variables were only useful when items were asked in all three years of the survey, to allow for valid comparisons across survey years.

We also evaluated models for individual or more finely grouped symptoms specifically dry eyes, itchy eyes, eye pain, blurred vision, sinus congestion, sinus pain, ear pain, ear drum rupture, ear infection, nosebleeds, runny nose, sore throat, ringing ears, combined EENT, cough, hoarseness/loss of voice, wheezing, lung infection symptoms, asthma symptoms, bronchitis symptoms, shortness of breath, combined lower respiratory, multiple chemical sensitivity symptoms, combined allergic symptoms, itchy/irritated skin, rash/hives, and combined dermatologic symptoms.

#### Exposure

The new type of uniform introduced to Alaska Airlines flight attendants in 2011 is the exposure of interest. The 2013 survey, which includes surveys through February 1, 2014, is representative of exposure to the new uniforms. Survey years 2007 and 2015 are used for comparison, with 2007 as the baseline, since both survey years reflect the Alaska Airlines flight attendants using other uniforms. The clothing materials in new and replacement uniforms can be found in the Appendix.

#### Covariates

Covariates in the models were age, gender, and current and past smoking.

### Statistical methods

We utilized STATA (Version 11.3, StataCorp, College Station, Texas) for descriptive statistics and data management, and we utilized SAS (Version 9.4, SAS Institute Inc., Cary, North Carolina) PROC GENMOD for statistical modeling. Inverse Probability Weighting was used to generate weights for each survey in the final Generalized Estimating Equations model of the odds of each symptom to account for the probabilities of attrition in the sampling frame. Separate logistic regression models were used to produce the fitted probabilities for return in 2013 and 2015 to implement inverse probability weighting. The study is observational and exploratory, so we did not correct for multiple testing.

## Results

The sample included 684 Alaska Airline flight attendants who completed a health survey in 2007 with varying survey completion rates in the follow-up years. The characteristics of the participant pool at each time period are listed in Table 1.

**Table 1:** Characteristics of survey participants by wave

Survey Year	2007	2013	2015	Total
	n (%)	n (%)	n (%)	n (%)
*N*	684	117	249	1050
Age (*n* = 1039)				
18–24 years old	11 (2)	0 (0)	0 (0)	11 (1)
25–34 years old	109 (15)	2 (2)	3 (1)	114 (11)
35–44 years old	195 (29)	11 (10)	28 (11)	234 (23)
45–54 years old	258 (38)	41 (37)	95 (39)	394 (38)
55–64 years old	105 (15)	43 (39)	95 (39)	243 (23)
65–74 years old	4 (1)	13 (12)	24 (10)	41 (4)
75 years or older	1 (0)	0 (0)	1 (0)	2 (0)
Gender (*n* = 1046)				
Female	573 (84)	98 (84)	203 (83)	874 (84)
Male	111 (16)	18 (16)	43 (17)	172 (16)
Overweight (*n* = 904)				
No	607 (91)	.	155 (68)	772 (85)
Yes	59 (9)	.	73 (32)	132 (15)
Current Smoker (*n* = 1020)				
No	662 (97)	103 (99)	226 (97)	991 (97)
Yes	22 (3)	1 (1)	6 (3)	29 (3)
Past Smoker (*n* = 1017)				
No	504 (74)	86 (83)	189 (83)	779 (77)
Yes	180 (26)	18 (17)	40 (17)	238 (23)
Tenure (*n* = 863)				
< 6 years	159 (25)	.	3 (1)	162 (19)
6–10 years	130 (21)	.	27 (11)	157 (18)
11–15 years	47 (8)	.	36 (15)	83 (10)
16–20 years	102 (16)	.	27 (12)	129 (15)
> 20 years	190 (30)	.	142 (61)	332 (38)
Education (*n* = 1009)				
High school graduate	39 (6)	6 (6)	15 (7)	60 (6)
GED	0 (0)	0 (0)	3 (1)	3 (0)
Some college, no degree	279 (41)	40 (38)	89 (39)	408 (40)
Two-year college degree	114 (16)	17 (16)	34 (15)	165 (16)
Four-year college degree	215 (32)	35 (34)	74 (33)	324 (33)
Graduate education	32 (5)	6 (6)	11 (5)	49 (5)

**Fig. 1:**
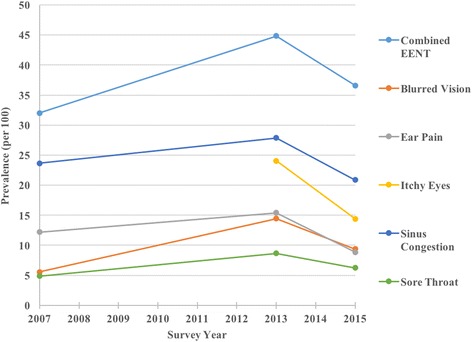
Plot of unweighted prevalence of EENT symptoms

**Fig. 2:**
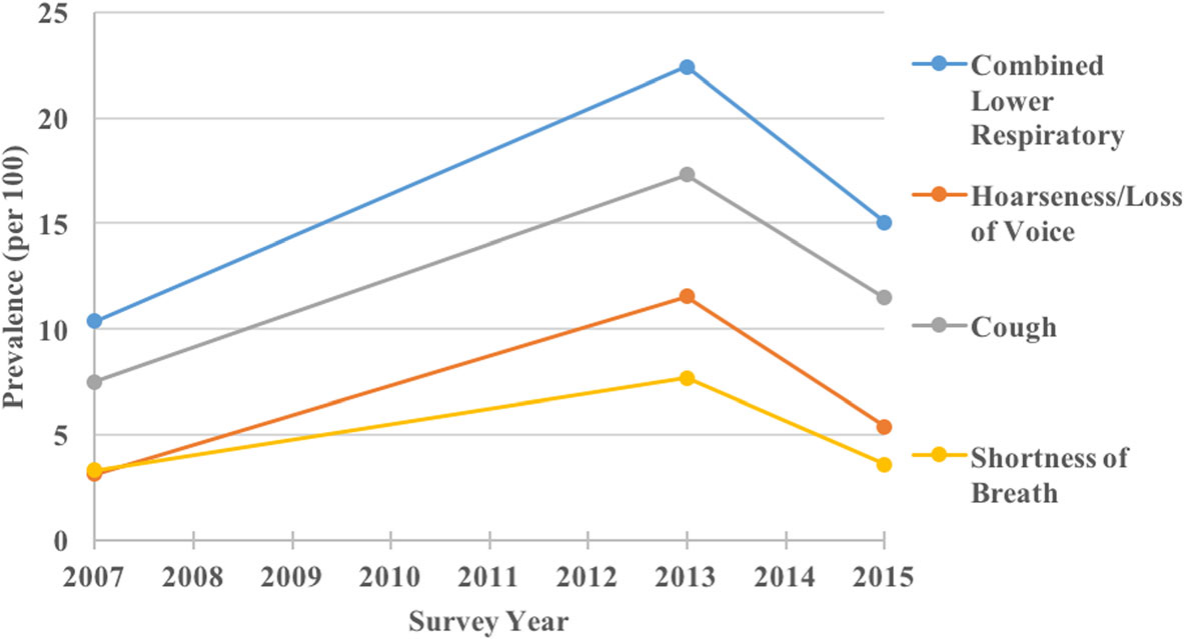
Plot of unweighted prevalence of lower respiratory symptoms

**Fig. 3:**
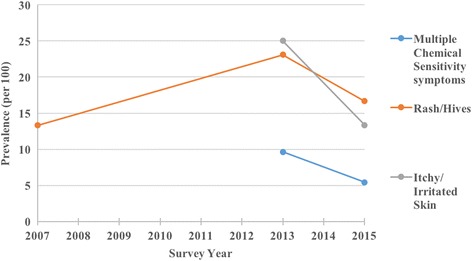
Plot of unweighted prevalence of skin and MCS symptoms

We calculated a standardized prevalence rate shown in the following Figures for several symptoms among the participants in each of the survey years. Note that missing entries occur in years when the survey question was not included. Each figure represents one of the four main symptom impact areas; EENT (Fig. 1), Lower Respiratory (Fig. 2), Skin and MCS (Fig. 3).

The prevalence of several symptoms appeared to spike in 2013, the year when the new uniforms were in use. The prevalence rate (per 100) increased for itchy eyes (24 vs 14), blurred vision (14 vs 6), sinus congestion (28 vs 24), ear pain (15 vs 12), sore throat (9 vs 5), cough (17 vs 7), hoarseness/loss of voice (12 vs 3), shortness of breath (8 vs 3), multiple chemical sensitivity symptoms (10 vs 5), itchy/irritated skin (25 vs 13), and rash/hives (23 vs 13).

To assess symptoms within individuals over time and to account for selection bias due to uneven follow-up among participants, we used Weighted Generalized Estimating Equations. For all analyses, we applied untrimmed inverse probability weights. A sensitivity analysis showed that using untrimmed weights lead to conservative *p*-values and results.

**Fig. 4:**
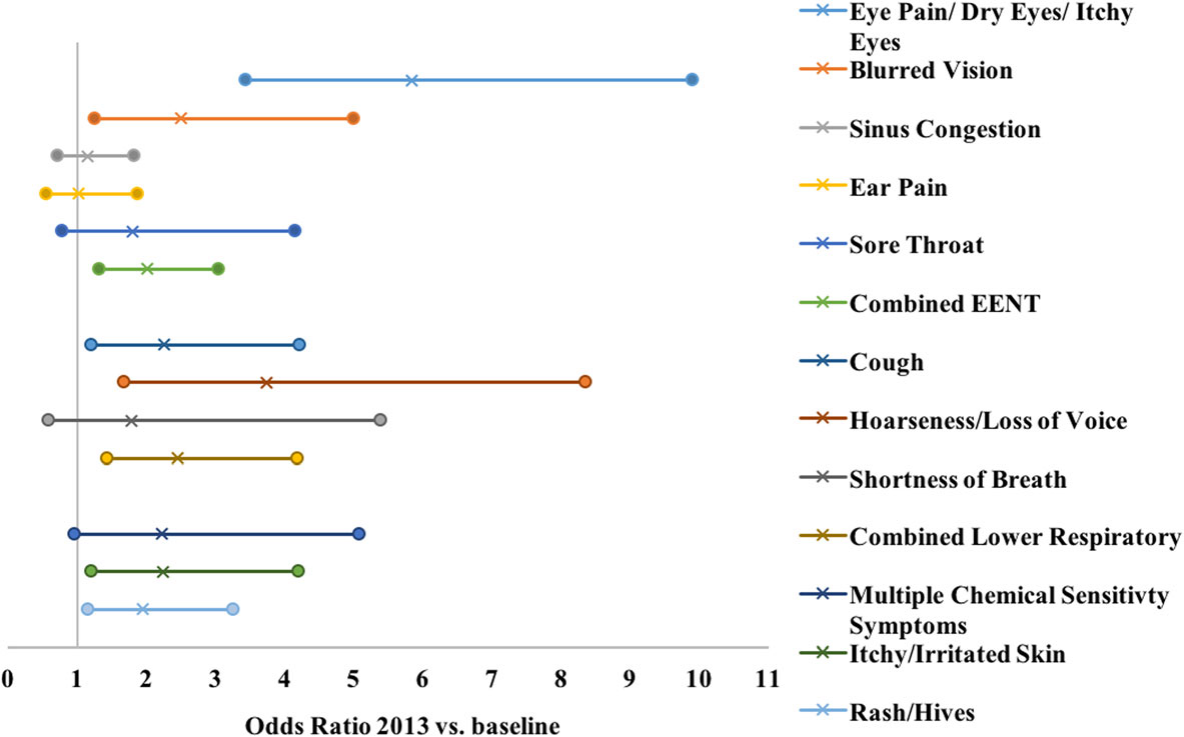
Plot of 2013 vs. baseline odds ratios in separate models. Odds ratios and 95% confidence intervals for the new uniform exposure year 2013 vs. baseline are shown for each outcome. Baseline is the exposure year 2007 for all outcomes, except multiple chemical sensitivity symptoms and itchy/irritated skin, for which 2015 is treated at baseline. Odds ratio estimates are denoted by “x” and 95% confidence limits are denoted by circles

Each symptom of interest was modeled separately and adjusted for sex, age, past smoking, and current smoking. We calculated odds ratios and 95% confidence intervals to test the strength of the associations. Fig. 4 displays the point estimates for the odds ratio for 2013 vs. baseline with 95% confidence intervals for each outcome of interest. Baseline is taken to be 2007, except for the model for multiple chemical sensitivity symptoms where 2015 is taken to be baseline because these symptoms were not included in the 2007 survey.

Figure 4 shows that all point estimates for the odds ratios are greater than 1. Outcomes with 95% confidence intervals excluding the null are: eye pain/dry eyes/itchy eyes (OR: 5.8; CI: 3.4, 9.9; *p* < 0.001), blurred vision (OR: 2.5; CI: 1.2, 5.0; *p* = 0.009), combined EENT (OR: 2.0; CI: 1.3, 3.0; *p* = 0.001), cough (OR: 2.3; CI: 1.2, 4.2; *p* = 0.011), hoarseness/loss of voice (OR: 3.7; CI: 1.7, 8.4; *p* = 0.001), combined lower respiratory (OR: 2.4; CI: 1.4, 4.2; *p* = 0.001), itchy/irritated skin (OR: 2.2; CI: 1.2, 4.2; *p* = 0.012), and rash/hives (OR 1.9; CI: 1.2, 3.3; *p* = 0.011). Non-significant outcomes include sinus congestion (OR: 1.1; CI: 0.7, 1.8; *p* = 0.562), ear pain (OR: 1.0; CI: 0.6, 1.9; *p* = 0.961), sore throat (OR: 1.8; CI: 0.8, 4.1; *p* = 0.163), shortness of breath (OR: 1.8; CI: 0.6, 5.3; *p* = 0.306), and multiple chemical sensitivity symptoms (OR: 2.2; CI: 0.9, 5.1; *p* = 0.061). Imprecise confidence intervals are associated with relatively low response rates for these questions.

Overall, the new uniforms are positively associated with specific health symptoms in our cohort of flight crew, with greater overall symptomology in 2013 as compared to baseline.

## Discussion

This study is the first to specify symptom prevalence rates in flight attendants after the introduction of new uniforms that generated numerous health complaints. Our investigation followed individuals over time to quantify the association between uniform exposures and symptoms occurring before, during and after the introduction of the suspect uniforms. We found that the introduction of the new flight attendant uniforms in Alaska Airlines was associated with increased EENT, lower respiratory, and skin symptoms.

The study relies on general health data collected as part of an on-going cohort of flight attendants. We accessed the cohort data retrospectively after the development of publicized uniform complaints. We expect, therefore, that any participant or researcher bias related to data collection with a specific hypothesis in mind was minimized. This opportunity to access a large cohort of flight attendants with exposure to the uniforms in question also permitted control for potentially confounding health variables such as age and smoking. The knowledge of uniform complaints circulating among flight attendants in this airline at the time may have raised awareness and therefore reporting of particular symptoms, but we believe this risk is small due to the more general purpose of the survey in which hundreds of data points were gathered.

Because of our retrospective design, we were unable to investigate other flight attendant health complaints potentially related to the new uniforms, such as symptoms of hair loss or thyroid dysfunction. Other studies have reported hair loss in relation to metals found in the textile dyes and thyroid dysfunction related to endocrine disruptors such as phthalates, also found in the uniforms [19–21]. Unfortunately, data about these conditions were not collected.

The survey data used for this analysis have several inherent limitations. First, all questions are answered by self-report and therefore our measures are subjective; there are no physician evaluations, medical records, or tests used to obtain health related information, which has likely led to misclassification of outcomes. The survey waves are also unevenly spaced, allowing for accumulated events or insidious problems unrelated to the exposure to be counted for the year 2013. However, the symptom questions in this survey are constrained by a seven-day window of experience and a visit to a care provider within the last year, thus, minimizing the problem of over-counting, and our analyses are adjusted for age, thus reducing the possibility of symptoms arising solely due to increasing age. Finally, our study was exploratory in nature, and we therefore did not correct for multiple testing.

Importantly, the exposure in this study is measured by proxy---according to time, and not according to specific chemical constituents. Although we have access to a number of chemical analyses from the airline, the manufacturer and laboratories commissioned by the union, the chemical compounds that can potentially be found in the materials are dependent on the different chemical panels included, the methods of analysis, and the materials tested. Importantly, the chemical compound concentrations varied by garment and material tested, and overall, these results tell us very little about the actual body burdens for any single chemical or mixture of contaminants or their possible health effects.

While harmful chemicals in clothing and furniture have lead to some product bans, labeling, and consumer protection rules (i.e. flame retardants, pthalates, formaldehyde) [22] chemicals in use are not routinely tested. Therefore, we generally know about harmful chemical exposures “after the fact” or once problems are revealed. It is also worth noting that similar uniform complaints have been raised among flight attendants of different airlines, most recently by American Airlines flight attendants in late 2016 [14].

In addition, even when testing is required in places like the European Union, testing given extreme environments or novel uses, or in the presence of other compounds, is not generally carried out. However, studies of aircraft cabin environments have found harmful compounds released from clothing and upholstery because of interactions with ozone in flight [23, 24]. Flight attendants are exposed to an unusual occupational environment, which includes various cabin air contaminants, Circadian rhythm disruption, high occupational noise, as well as changes in pressure, oxygenation, and humidity [25], and it is unclear how all of these factors come together to affect potential reactions to chemical compounds in clothing.

Our study findings are important not only for flight attendants. An occupational cohort sometimes makes visible the magnitude of harmful exposures by the sheer volume of complaints in a defined population that may be otherwise invisible or interpreted as individual sensitivity. Our findings raise the question of whether flight attendant symptoms experienced in relation to the introduction of new uniforms may suggest a potential problem for the population at large in regard to chemicals in our clothes.

Unfortunately, we know little about chemicals in our clothing as compared to substances we ingest, even though skin absorption can be quite efficient, as we know from new drug delivery mechanisms. Further, in the case of many chemicals, threshold levels for health effects are based on a few animal studies [26], as opposed to large randomized clinical trials. In addition, relatively few chemicals of concern in clothing have been tested in laboratories. Besides metals and dyes, important clothing contaminants are formaldehyde and formaldehyde releasers, dioxin, perfluorinated compounds, flame retardants, phthalates and other plasticizers such as diisodecyclmaleate, and pesticides used in shipment [27–30]. These and other chemicals have been associated with serious health consequences in laboratory and epidemiologic studies [31, 32].

In summary, additional analyses of the chemical compounds in flight attendant uniforms that are needed include modeling exposures based on concentrations, chemical binding, dispersions with sweat and friction, skin absorption and chemical reactions and breakdown due to concomitant exposures such as UV radiation and ozone chemistry aboard the aircraft. The exposure of chemical contaminants in textiles need much further study particularly in regard to body burdens and safe concentrations under conditions of use.

## Conclusions

This study was not designed to identify specific harmful compounds or to estimate dose–response thresholds. Rather, the purpose of this study was to provide information regarding the likelihood that textile exposures may be associated with health effects among flight crew.

We found that the introduction of new flight attendant uniforms was associated with quantifiable health effects. More specifically, we found the new uniform introduction to be related to skin rashes and itchiness, as well as respiratory and allergic symptoms. Our study is the first to examine the relationship between uniforms containing harmful chemical compounds and health outcomes, and our flight attendant cohort allowed us the opportunity to do so in a rigorous way. These results raise the seriousness of the flight attendant complaints about potential harms and the need for further investigation.

## Appendix

**Table 2:** Clothing materials in new and replacement uniforms*

Garment	New Uniforms 2011–14	Replacement Uniforms 2014–17
Skirt	50% wool, 48% polyester, 2% spandex (lining = 100% polyester) - assembled in Indonesia	51% polyester, 42% wool, 7% elastane (lining = 100% polyester) - assembled in Sri Lanka
Blazer	53% polyester, 43% wool, 4% elastane (lining = 100% polyester) - assembled in China	52% polyester, 42% wool, 6% spandex (lining = 100% polyester) - assembled in Sri Lanka
Shirts	60% cotton, 40% polyester - assembled in Indonesia	55% cotton, 45% polyester - assembled in Indonesia
Cardigan/Sweater	55% acrylic, 23% wool, 22% nylon - location of assembly unknown (a wool-free acrylic/nylon alternative was also available) 53% polyester, 43% wool, 4% elastane (lining = 100% polyester) - assembled in China	55% cotton, 45% polyester - assembled in Indonesia
Pants	53% polyester, 43% wool, 4% elastane (lining = 100% polyester) - assembled in China	52% polyester, 42% wool, 6% spandex (lining = 100% polyester) - assembled in Sri Lanka 52% polyester, 42% wool, 6% spandex (lining = 100% polyester) - assembled in Sri Lanka

*Material information taken from flight attendant clothing labels
